# Bone marrow mesenchymal stem cell-derived exosomes prevent osteoarthritis by regulating synovial macrophage polarization

**DOI:** 10.18632/aging.104110

**Published:** 2020-12-22

**Authors:** Jiyong Zhang, Yuluo Rong, Chunyang Luo, Weiding Cui

**Affiliations:** 1 Department of Orthopaedic, The First Affiliated Hospital of Nanjing Medical University, Nanjing, Jiangsu, China; 2 Department of Orthopaedic, The First People's Hospital of Wuhu, Wuhu, Anhui, China

**Keywords:** osteoarthritis, bone marrow mesenchymal stem cells, exosomes, synovial macrophages, polarization

## Abstract

Osteoarthritis is a chronic degenerative disease that can lead to restricted activity or even disability. Bone marrow mesenchymal stem cells can repair cartilage damage and treat osteoarthritis via cell therapies or in-tissue engineering. Research has shown that the paracrine mechanism is the main mode of action of mesenchymal stem cells. Exosomes are the smallest known membrane-bound nanovesicles. Exosomes are also important carriers of paracrine delivery agents and promote communication between cells. We demonstrated that bone marrow mesenchymal stem cell-derived exosomes can delay the progression of osteoarthritis. Exosomes alleviate cartilage damage, reduce osteophyte formation and synovial macrophage infiltration, inhibit M1 macrophage production and promote M2 macrophage generation. In synovial fluid, the expression levels of the proinflammatory cytokines, IL-1β, IL-6, and TNF-α were decreased and the release of the anti-inflammatory cytokine, IL-10 was increased. *In vitro*, macrophages treated with exosomes maintain chondrocytes’ chondrogenic characteristics and inhibit hypertrophy. Our results demonstrated that bone marrow mesenchymal stem cell-derived exosomes may relieve osteoarthritis by promoting the phenotypic transformation of synovial macrophages from M1 to M2.

## INTRODUCTION

Osteoarthritis (OA) is a degenerative joint disease that seriously affects cartilage and surrounding tissues [1]. The pathogenesis of OA results in the degeneration of chondrocytes and cartilage. Imbalances in the synthesis of articular chondrocytes, extracellular matrix, and subchondral bone is a pathological feature during degradation [2]. Articular cartilage is a component of the articular surface that acts as a buffer, allowing fluid flow and the dispersion of pressure. After the degeneration of cartilage, degradation products cause inflammation in the joints, which are accompanied by changes in subchondral bone and the synovium. At present, the clinical treatment of early OA includes the use of anti-inflammatory analgesic drugs, the application of chondroprotective drugs, and arthroscopic debridement, among other approaches [3]. However, those measures can only alleviate the clinical symptoms and cannot prevent the development of disease; thus, the effect is not satisfactory. Advanced OA is often treated with artificial joint replacement, which can relieve pain and restore patients’ daily living abilities. However, complications such as the necessity to wear prostheses and loosening of artificial joints greatly limit the application of artificial joint replacement [4]. The incidence of OA in the middle-aged population can be as high as 40%-80%, and the disability rate is greater than 50% [5], which imparts a substantial burden to individuals and society. Therefore, to determine the pathogenesis of OA, effective intervention in the early stages of pathogenesis is a key step for successful treatment.

Synovial inflammation is associated with the pathogenesis of OA and is significantly associated with the severity of OA [6–8]. The synovial membrane is thin, soft, loose connective tissue that is coated on the inner side of the joint capsule and forms a closed capsule around the joint cavity. The normal synovium can be divided into an intimal lining layer and a synovial sub-lining layer. The intimal lining is composed of two to three layers of synovial cells, including macrophage-like cells, fibroblast-like cells, dendrite-like synoviocytes, and a few mesenchymal stem cells. The synovial sub-lining layer consists of blood vessels, fibrous connective tissue, macrophages, and a small number of lymphocytes [9, 10]. Synovitis is closely related to the polarization of synovial macrophages. The main features of synovitis are synovial tissue hyperplasia, inner macrophage aggregation, and cell secretory dysfunction [10]. Macrophages can be divided into two polarization states of classically activated M1 macrophages and alternatively activated M2 macrophages under different microenvironments [11, 12]. After being stimulated by lipopolysaccharide (LPS) and interferon-γ, M1 macrophages secrete a large number of pro-inflammatory cytokines, such as interleukin-1β, IL-6, and TNF-α, triggering the body's inflammatory response. However, excessive inflammation can cause damage to normal tissues of the body [13, 14]. M2 macrophages have anti-inflammatory activities and mainly secrete anti-inflammatory cytokines, such as IL-4, IL-10 and TGF-β, which can inhibit the development of inflammation and promote wound healing [14]. Studies have shown that synovial macrophage polarization is closely related to the development of OA. Zhang et al. [15] found that in a mouse OA model, macrophages in the synovial membrane and joint cavity aggregated, M1 synovial macrophages increased and accelerated OA progression.

Bone marrow mesenchymal stem cells (BMSCs) have the potential for multi-directional differentiation and self-renewal, and can differentiate into cells such as bone, cartilage, muscle, and fat [16]. BMSCs are involved in immune regulation, inhibition of inflammation, secretion of various cell growth factors, and tissue repair [17]. Thus, MSCs are a seed cell commonly used for clinical cell therapy and tissue engineering research. Some progress in MSC-based OA treatment strategies have been achieved. An increasing number of studies have confirmed that the paracrine mechanism is the primary function of BMSCs and BMSC-derived exosomes are one of the important ways for MSCs to exert therapeutic effects [4]. Exosomes have a vesicular structure of between 30 and 150 nm in diameter, and are secreted by most cells into body fluids [18]. Exosomes contain a variety of components, including proteins, DNA, messenger RNA, microRNA, and long non-coding RNA, which play important roles in intercellular communication [19]. BMSC-derived exosomes have immunoregulatory functions, can regulate innate and adaptive immune responses, inhibit excessive inflammation, and promote tissue repair [20]. BMSC-derived exosomes can also act on macrophages, thereby promoting the polarization of M2 macrophages and increasing the secretion of anti-inflammatory cytokines. Zhao et al. [21] found that MSCs-derived exosomes from infrapatellar fat pads can promote the conversion of macrophages to the M2 phenotype, which express high levels of Arg-1 and IL-10, and inhibit the inflammatory response. However, there are few reports of the association of BMSC-derived exosomes with macrophage polarization in an OA model.

In this study, we hypothesized that BMSC-derived exosomes relieve OA by promoting the phenotypic transformation of synovial macrophages from M1 to M2. We prepared BMSC-derived exosomes and treated a rat OA model by intra-articular injection. We found that cartilage damage, osteophyte formation and synovial macrophage infiltration was reduced in treated rats. Additionally, we found that macrophages were also transformed from M1 to M2, and the levels of inflammatory cytokines were decreased in synovial fluid. We conducted experiments *in vitro* to further verify that BMSC-derived exosomes maintain chondrogenic characteristics and inhibit hypertrophy of chondrocytes by promoting the transformation of synovial macrophages from M1 to M2. These results provide a new avenue for the treatment of OA using BMSC-derived exosomes.

## RESULTS

### Identification of BMSCs

**Figure 1:**
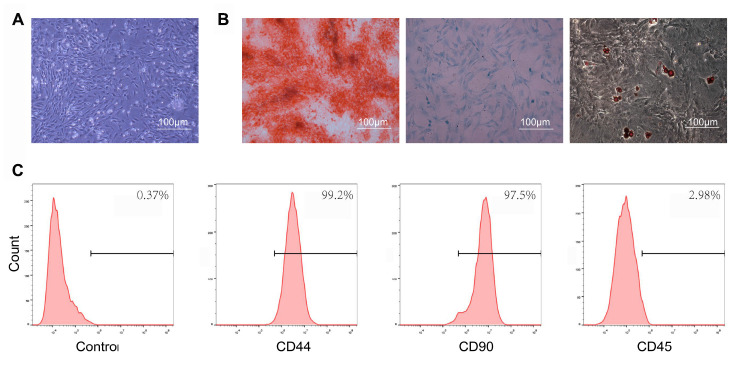
**Characterization of bone marrow mesenchymal stem cells (BMSCs).** (**A**) BMSCs exhibited a representative spindle-like morphology. Scale bar: 100 μm. (**B**) BMSCs showed osteogenic pluripotent differentiation abilities, adipogenesis, and cartilage formation. Scale bar: 100 μm. (**C**) Flow cytometry analysis of characteristic cell surface markers of BMSCs. The mesenchymal markers, CD44 and CD90, were positive, while the hematopoietic cell surface marker, CD45, was negative.

In the present study, we isolated BMSCs that were of irregular shape, spindle shape, and polygon in the bright field images (Figure 1A). The results of alizarin red, oil red O and Alimin Blue staining confirmed that BMSCs had multi-directional differentiation potentials, including differentiation into osteoblasts, adipocytes and chondrocytes (Figure 1B). BMSCs were identified by flow cytometry, and were positive for the mesenchymal markers CD44 and CD90, while they were negative for the hematopoietic cell surface marker, CD45 (Figure 1C).

### Characterization of BMSC-derived exosomes

**Figure 2:**
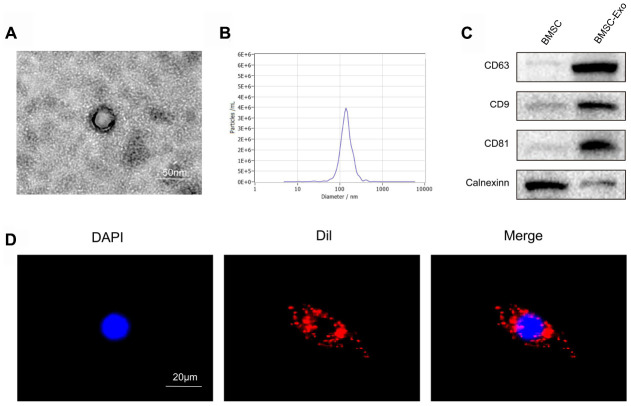
**Characterization and uptake of BMSC exosomes.** (**A**) Transmission electron microscopy (TEM) showed that BMSC-derived exosomes had a typical cup-like morphology. Scale bar: 50 nm. (**B**) Nanosight Tracking Analysis (NTA) detection revealed that the average vesicle diameter was about 140 nm. (**C**) Western blot analysis of exosome lysates revealed positive expression of the exosome surface markers, CD63, CD81 and CD9. No cerulein was detected in BMSC-derived exosomes. (**D**) Detection of exosome uptake by BMSCs. Dil : red, DAPI: blue. Scale bar: 20 μm.

Exosomes were isolated from the supernatant of BMSCs by ultrafiltration and centrifugation. Purified BMSC-derived exosomes were identified by NTA, transmission electron microscopy and western blotting. Transmission electron microscopy showed that BMSC-derived exosomes showed a typical cup-like morphology (Figure 2A). NTA detection revealed that the average vesicle diameter was about 140 nm (Figure 2B). Western blot analysis of exosome lysates revealed positive expression of the exosomal surface markers CD63, CD81 and CD9, while no cerulein was detected (Figure 2C). Collectively, these results confirm the successful separation of exosomes from the supernatant of BMSCs.

**Figure 3:**
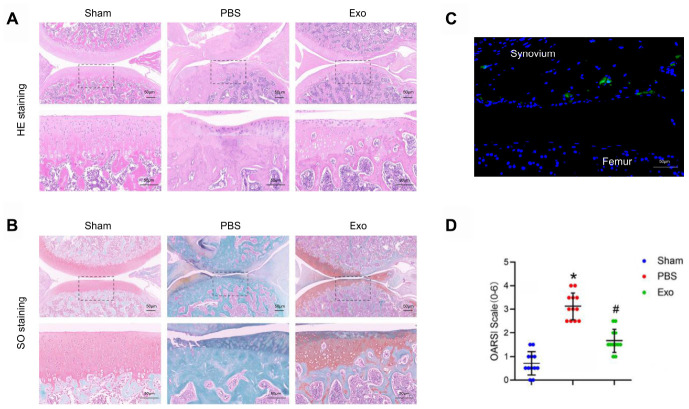
**BMSC exosomes prevented cartilage destruction in a rat model.** (**A**) Representative images of HE staining in knee sections. Scale bar: 50 μm. (**B**) Representative images of Safranin O / Fast Green staining in knee sections. Scale bar: 50 μm. (**C**) Representative adoptive Dil-labelled BMSC-Exos (green fluorescence) in a knee joint. Scale bar: 50 μm (**D**) Evaluation of cartilage destruction using the OARSI scoring system. *p < 0.05 compared to the Sham group; # p < 0.05 compared to the PBS group.

To investigate the feasibility of using BMSC-derived exosomes to treat OA, we examined the cellular uptake of Dil-labeled exosomes (Dil-exosomes). After RAW264.7 cells were incubated with Dil-exosomes for 24 h, Dil-exosomes were observed in the cytoplasm by fluorescence microscopy to confirm uptake (Figure 2C). *In vivo* experiments further demonstrated that Dil-exosomes could accumulate in the knee joint and be taken up by synovial cells following injection (Figure 3C).

### BMSC-derived exosomes relieve osteoarthritis

**Figure 4:**
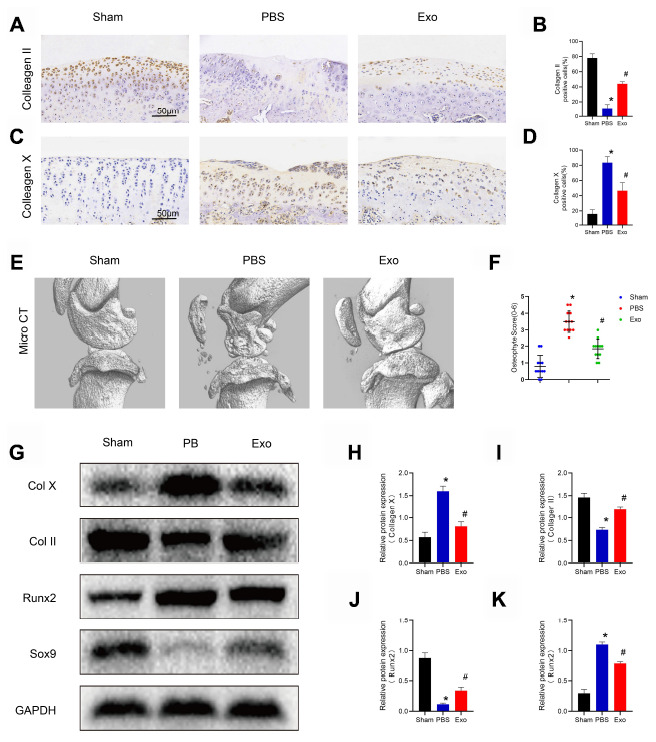
**BMSC exosomes inhibited chondrocyte hypertrophy and reduced osteophyte formation in the rat model.** (**A**) and (**B**) Immunohistochemistry and analysis of collagen II in knee sections. Scale bar: 50 μm. (**C**) and (**D**) Immunohistochemistry and analysis of collagen X in knee sections. Scale bar: 50 μm. (**E**) Micro-CT scan and three-dimensional reconstruction of the knee joint in each experimental group. (**F**) Osteophyte score and quantitative analysis of the volume of the region of interest (ROI). (**G**–**K**) Western blot results and analyses of cartilage tissue. *p < 0.05 compared to the Sham group; # p < 0.05 compared to the PBS group.

MSC-derived exosomes isolated from various tissue sources have potential therapeutic benefits in many diseases [22–24], including cartilage damage and repair. We hypothesized that BMSC-derived exosomes can alleviate OA. In this study, in order to evaluate the therapeutic effect of BMSC-derived exosomes in OA, intra-articular injections were performed with PBS or BMSC-derived exosomes at 4 weeks postoperatively. At the 8^th^ week after operation, the PBS group showed severe cartilage degradation, markedly thinned articular cartilage, increased surface fibrosis area, abnormal distribution of chondrocytes (Figure 3A and 3B), decreased expression of type II collagen, which is the main component of hyaline cartilage, and increased expression of type X collagen, which is the main component of fibrocartilage (Figure 4A–4D). Micro CT analysis and three-dimensional (3D) modeling of the knee showed a significant increase in the number and surface area of the osteophyte around the joint (Figure 4E and 4F). In contrast, in the exosomes group, cartilage was less degraded, chondrocytes maintained near-normal morphology and distribution, and the number and surface area of osteophytes around the joints were less than those in the PBS group. The OARSI score was significantly lower in the exosomes group (Figure 3D). The western blot results of cartilage tissue showed that compared with the exosomes group, the expression of the hypertrophic gene, collagen X, and runx2 was decreased. The expression of the chondrogenic genes, collagen II and sox9, was also increased (Figure 4G–4K). These findings indicated that BMSC-derived exosomes inhibit cartilage degradation and osteophyte formation during OA progression in rats.

### BMSC-derived exosomes change the M1/M2 subsets of synovial macrophages

**Figure 5:**
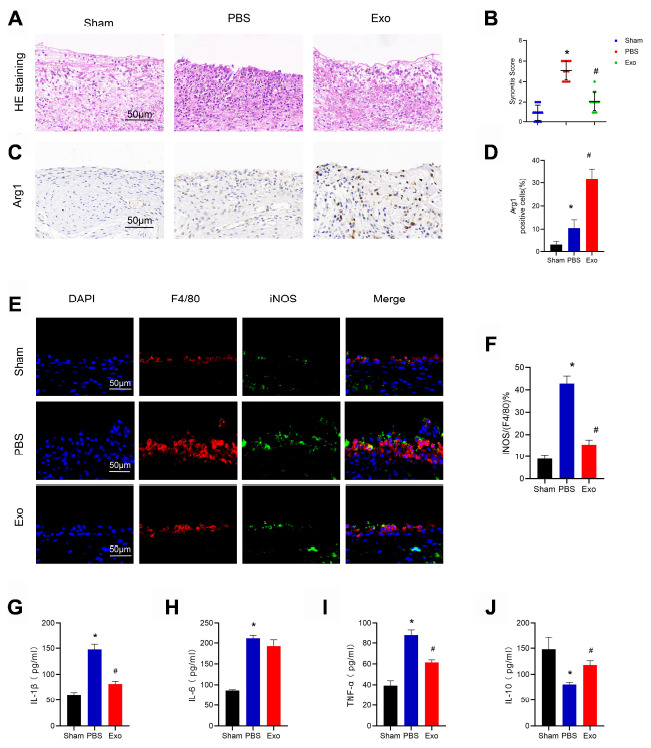
**BMSC exosomes reduced synovial macrophage infiltration, induced changes in M1/M2 subsets, and reduced inflammation in the rat model.** (**A**) Representative images of HE staining in synovial tissue. Scale bar: 50 μm. (**B**) Quantification of synovitis scores in synovial tissue. (**C**) and (**D**) Immunohistochemistry and analysis of Arg1 in synovial tissue, respectively. Scale bar: 50 μm. (**E**) and (**F**) Immunofluorescence and analysis of F4/80 and iNOS, respectively, in synovial tissue. Scale bar: 50 μm. (**G**–**J**) Determination of the cytokines, IL-1β, IL-6, TNF-α, and IL-10 in synovial fluid from each test group. * p < 0.05 compared to the Sham group; # p < 0.05 compared to the PBS group.

As mentioned previously, M1 macrophages increase the inflammatory response during OA. In this study, we found that BMSC-derived exosomes can alleviate the progression of OA. However, whether exosomes promote the phenotypic transformation of synovial macrophages from M1 to M2 is unknown. To address this question, knee joint sample sections were used for immunohistochemistry and immunofluorescence to detect changes in the synovial M1 / M2 subsets. Compared with the sham group, synovial hyperplasia and cell infiltration were significantly increased in the PBS group, and the synovitis score was significantly increased (Figure 5A and 5B). F4/80+ (macrophage marker) -positive cells in synovial tissue were significantly increased, and the ratio of F4/80+iNOS+ (M1-like macrophage markers) positive cells also increased significantly (Figure 5E and 5F). However, Arg1+ (M2-like macrophage markers) increased slightly (Figure 5C and 5D). Compared with the PBS group, synovial hyperplasia and cell infiltration were reduced in the Exo group, the ratio of M1-positive cells was significantly decreased, and the ratio of M2-positive cells was increased. ELISA detection of synovial fluid showed that the levels of the pro-inflammatory cytokines IL-1β, TNF-α were significantly decreased in the Exo group, while the level of the anti-inflammatory cytokine IL-10 was increased (Figure 5G–5I). These findings indicate that BMSC-derived exosomes change the M1/M2 subsets of synovial macrophages during OA progression in rats and decrease the level of inflammation in synovial fluid.

### BMSC-derived exosomes promote the phenotypic transformation of macrophages from M1 to M2 *in vitro*

**Figure 6:**
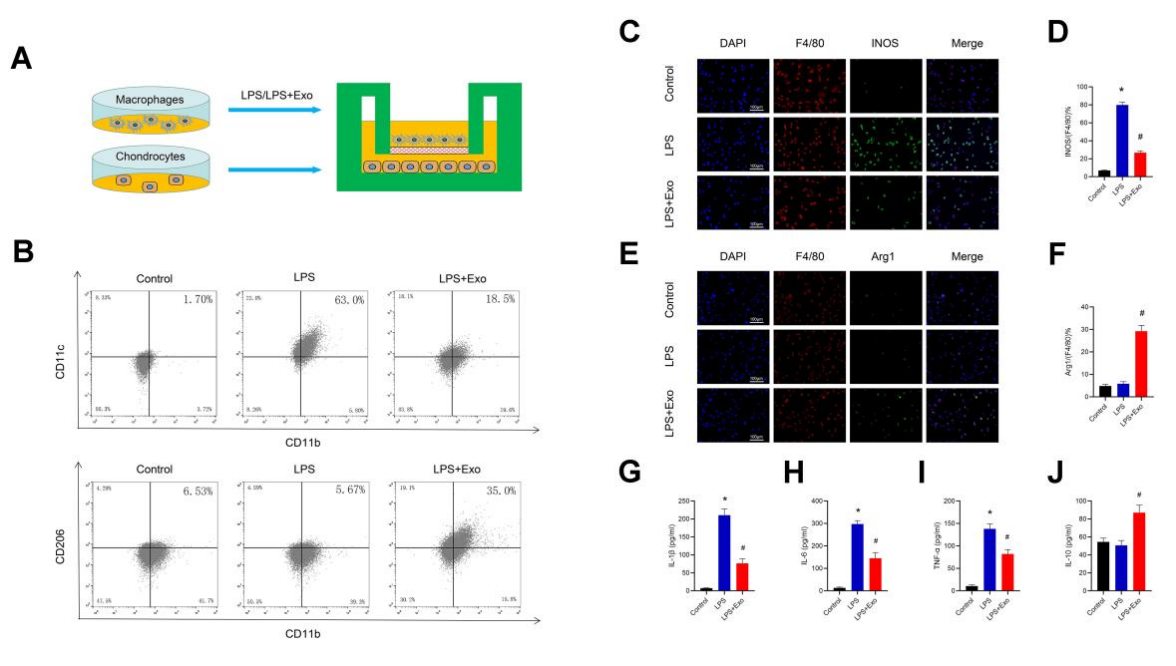
**BMSC exosomes promote the phenotypic transformation of macrophages from M1 to M2 and reduce inflammation.** (**A**) The protocol of the co-culture system. (**B**) Detection of CD11c (M1 marker) and CD206 (M2 marker) expression in RAW264.7 cells by flow cytometry. (**C**) and (**D**) Detection of INOS (M1 marker) by immunofluorescence in RAW264.7 cells and statistical results. Scale bar: 100 μm. (**E**) and (**F**) Detection and analysis of Arg-1 (M2 marker) in RAW264.7 cells by immunofluorescence and statistical results. Scale bar: 100 μm. (**G**–**J**) Determination of the cytokines, IL-1β, IL-6, TNF-α and IL-10 in supernatants. *p < 0.05 compared to the Control group; # p < 0.05 compared to the LPS group.

We further verified the regulation of BMSC-derived exosomes on macrophage polarization *in vitro*. LPS was used to induce RAW264.7 polarization to the M1 phenotype. Cells were incubated with LPS (50 ng/mL) or LPS (50 ng / ml) + Exo (1 μg/mL) for 24 h, and then the cells and supernatant were collected for assaying. Flow Cytometry and immunofluorescence were used to detect macrophage typing, and ELISA was used to determine the cytokine content in the supernatant. Immunofluorescence results showed that the proportion of M1 macrophages (F4/80+iNOS+) was significantly up-regulated after LPS treatment, while the proportion of M2 macrophages (F4/80+Arg1+) was not noticeably different. Compared with the LPS group, the M1 ratio of the exosomes-treatment group was significantly decreased, while the M2 ratio was increased (Figure 6C–6F). The results of flow cytometry experiments showed that the proportion of CD11c+CD11b+(M1) in the Exo-treatment group was significantly decreased, while the proportion of CD206+CD11b+(M2) was increased (Figure 6B). The ELISA results in the supernatant showed that the levels of the pro-inflammatory cytokines IL-1β, IL-6, TNF-α were significantly decreased in the exosomes-treatment group, while the levels of the anti-inflammatory cytokine IL-10 was increased (Figure 6G–6J). Those data indicate that BMSC-derived exosomes promote the phenotypic transformation of macrophages from M1 to M2 *in vitro* and reduce inflammation.

### Transformation of macrophage polarization maintains chondrogenic characteristics and inhibits hypertrophy of chondrocytes

**Figure 7:**
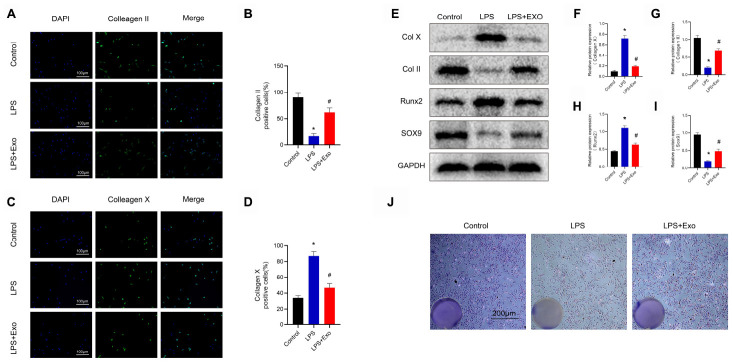
**Macrophages treated with BMSC exosomes maintain chondrogenic characteristics and inhibit hypertrophy of chondrocytes.** (**A**) and (**B**) Detection of the expression of collagen II by immunofluorescence in chondrocytes and statistical results. Scale bar: 100 μm. (**C**) and (**D**) Detection of the expression of collagen X by immunofluorescence in chondrocytes and statistical results. Scale bar: 100 μm. (**E**–**I**) Detection of the expression of collagen II, collagen X, SOX9 and Runx2 in chondrocytes by western blotting and statistical results. (**J**) Image of toluidine blue staining of chondrocytes. Scale bar: 200 μm. *p < 0.05 compared to the Control group; # p < 0.05 compared to the LPS group.

In order to investigate whether changes in macrophage polarization have an effect on chondrocytes, we co-cultured macrophages with chondrocytes for 72 h, and then analyzed the chondrocytes (Figure 6A). The results of immunofluorescence assays showed that compared with the control group, the collagen X gene of the cartilage hypertrophy in the LPS-treatment group was significantly up-regulated, while the proportion of the collagen II gene was decreased (Figure 7A–7D). Compared with the LPS group, the expression of collagen X in the Exo-treatment group was significantly decreased, while that of collagen II was significantly increased. Western blot results showed that compared with the LPS group, the expression of collagen X and runx2 were decreased, while the expressions of collagen II and sox9 were increased (Figure 7E–7I). The results of toluidine blue staining showed the markedly increased expression of GAG in chondrocytes cultured with Raw264.7 cells induced by LPS and exosomes (Figure 7J). These data indicate that the phenotypic conversion of macrophages from M1 to M2 maintain chondrogenic characteristics and inhibit hypertrophy of chondrocytes.

## DISCUSSION

In this study, we found that intra-articular injection of BMSC-derived exosomes promoted the transformation of synovial macrophages from M1 to M2, inhibited the release of inflammatory cytokines, reduced the infiltration of synovial inflammatory cells and the damage of articular cartilage, and delayed the progression of OA. In order to verify this conclusion, we further conducted *in vitro* experiments, using BMSC-derived exosomes to treat LPS-induced RAW264.7. We found that BMSC-derived exosomes can promote the conversion of RAW264.7 from M1 to M2, inhibiting the expression of inflammatory cytokines, enhanced the expression of anti-inflammatory cytokines. In addition, we found that BMSC-derived exosomes can inhibit chondrocyte hypertrophy and degeneration caused by M1 macrophages. These data indicate that BMSC-derived exosomes treats OA in part by promoting conversion of macrophages from M1 to M2.

During the development of OA, the inflammatory cytokines IL-1β, IL-6 and TNF-α, as well as the catabolic factors such as matrix metalloproteinase MMP-13 are rapidly up-regulated, accelerating chondrocyte apoptosis and matrix degradation [25]. Inhibition of inflammation is an important part of preventing the development of OA, while the synovial macrophage phenotype is closely related to inflammation. For example, it has been reported that M1 macrophages in OA synovial tissue inhibit MSC chondrogenic differentiation by IL-6 *in vitro* [26], whereas M2 macrophages inhibit inflammation by producing IL-10, which supports the survival of cartilage grafts [27]. In our study, BMSC-derived exosomes attenuated the infiltration of synovial macrophages, promoted the phenotypic conversion from M1 to M2, and reduced the levels of inflammation in the OA rat model. In this study, we demonstrated that BMSC-derived exosomes can promote the conversion of RAW264.7 from MI to M2, reduce the expression of the proinflammatory cytokines IL-1 B, TNF-a and IL-6, and increase the expression of the anti-inflammatory factor, IL-10. To further investigate the effect of changes in macrophage polarization on chondrocytes, we co-cultured pre-treated RAW264.7 with chondrocytes and found that M2 type RAW264.7 cells enhanced the expression of the chondrogenic genes, collagen II and sox9. Co-culturing also inhibited the expression of the hypertrophic genes, collagen X and runx2. Because the experimental application is an indirect co-culture method, the regulatory effect of RAW264.7 through direct cell contact needs further investigation.. This is consistent with the function of embryonic-derived mesenchymal stem cell exosomes described by Zhang et al [28]. In that study, MSC-derived exosomes attenuated the expression of pro-inflammatory factors (IL-1b, IL-6 and TNF-α) and induced the expression of anti-inflammatory factors (IL-10 and TGF-β1) in an osteochondral defect model.

Studies have shown that MSC-derived exosomes regulate macrophage polarization in many fields, thus, exhibiting an anti-inflammatory effect of immune regulation. Studies by Ni et al. [29] showed that BMSC-derived exosomes can inhibit early neuroinflammation in mice with traumatic brain injury by regulating the polarization of microglia / macrophages; thus, playing a neuroprotective role. Sun et al. [30] showed that hucMSC-derived exosomes promote the conversion of bone marrow-derived macrophages from M1 to M2, reduce inflammation at the site of injury, and promote the healing of spinal cord injuries. Studies by Deng et al. [31] showed that ADSC-derived exosomes improve cardiac damage after myocardial infarction by activating S1P / SK1 / S1PR1 signaling and promoting M2 macrophage polarization. Studies by He et al. [32] showed that miR-223 targeting exosomes derived from BMSCs can target pknox1 to promote the polarization of M2 macrophages and accelerate the healing of skin wounds.

In this study, we demonstrated that BMSC-derived exosomes can promote the conversion of RAW264.7 from M1 to M2, reduce the expression of the proinflammatory cytokines IL-1β, TNF-α and IL-6, and increase the expression of the anti-inflammatory factor, IL-10. To further investigate the effect of changes in macrophage polarization on chondrocytes, we co-cultured pre-treated RAW264.7 with chondrocytes and found that M2 type RAW264.7 cells enhanced the expression of the chondrogenic genes, collagen II and sox9. Co-culturing also inhibited the expression of the hypertrophic genes, collagen X and runx2. Because the experimental application is an indirect co-culture method, the regulatory effect of RAW264.7 through direct cell contact needs further investigation.

BMSC-derived exosomes can repair cartilage damage and delay OA through a variety of mechanisms, including promoting chondrocyte proliferation, promoting extracellular matrix secretion, inhibiting chondrocyte apoptosis, and maintaining chondrocyte homeostasis. In this study, BMSC-derived exosomes promoted the transformation of synovial macrophages from M1 to M2, reducing the infiltration of synovial inflammatory cells. In addition, BMSC-derived exosomes reduced the expression of the pro-inflammatory cytokines, IL-1β and TNF-α in joint fluid, increased the expression of the anti-inflammatory cytokine, IL-10, inhibited the inflammatory response, reduced cartilage damage, and delayed the progression of OA. BMSC-derived exosomes are rich in biologically-active substances, including proteins, lipids, and nucleic acids, among others [33]. Among such substances, a large number of miRNAs regulate multiple signal transduction pathways. Tao et al. [34] showed that overexpression of miR-140-5p synovial mesenchyme indicates that the exosomes of stem cells enhances the proliferation and migration of chondrocytes, while avoiding damage to extracellular matrix secretion and inhibiting the progression of OA. Wu et al. [35] found that exosomes derived from mesenchymal stem cells in subpatellar fat pads can inhibit the mTOR signaling pathway through miR-100-5p, enhance autophagy, inhibit apoptosis, promote extracellular matrix synthesis, and alleviate the progression of OA. Mao [36] and other researches have shown that BMSC-derived exosomes overexpressing miR-92a-3p can inhibit the expression of WNT5A in chondrocytes, promote the proliferation of chondrocytes as well as their secretion, and inhibit the degradation of cartilage. In this study, we only initially demonstrated that BMSC-derived exosomes delayed OA by regulating macrophage polarization. In subsequent research, we plan to further explore the key substances (such as miRNAs) in BMSC-derived exosomes for the treatment of osteoarthritis.

## CONCLUSIONS

In conclusion, BMSC-derived exosomes promoted the phenotypic transformation of macrophages from M1 to M2, reduced inflammatory cytokines, and promoted the release of anti-inflammatory cytokines. Intra-articular injection of BMSC-derived exosomes reduced inflammation, reduce cartilage damage, and inhibited OA progression. Therefore, this study may provide new avenues for the clinical treatment of BMSC-derived exosomes in OA.

## MATERIALS AND METHODS

### Isolation and culture of cells

All animal experiments were performed in accordance with the Animal Research Committee of Nanjing Medical University. Rats were euthanized by CO_2_ inhalation, the knee joint was separated, the soft tissue around the joint was removed, and the cartilage tissue was cut into pieces for use. The femur and sacral cavity were rinsed repeatedly, the rinsing solution was collected, the pellet was resuspended after centrifugation and inoculated in Dulbecco's Modified Eagle’s Medium/Nutrient Mixture F-12 medium (DMEM/F-12, Gibco, USA) containing 10% fetal bovine serum (FBS, Gibco, USA) and 1% cyan-streptomycin (P/S, Sigma, USA) at 37° C in humid air with 5% CO_2_. Cartilaginous tissue was washed three times with phosphate buffered saline (PBS, Gibco, USA) and digested with 0.2% type II collagenase (Sigma-Aldrich, USA) at 37° C for 6-8 h. The pellet was resuspended after centrifugation and inoculated in high-glucose Dulbecco's Modified Eagle’s Medium (HG-DMEM, Gibco, USA) containing 10% FBS and 1% P/S at 37° C in humid air with 5% CO2. All medium was replaced every 3 days and passaging was performed when 80%~90 of cells is merged.

### Identification of BMSCs

For surface markers, five-passage BMSCs were used with flow cytometry to identify the mesenchymal markers, CD44 and CD90, and the hematopoietic cell surface marker, CD45. For multidirectional differentiation potential, osteogenic induction medium, adipogenic induction medium and chondrogenic induction medium (all from Cyagen, China) were used to replace the complete medium to induce differentiation. The process of inducing differentiation was performed as described in the manufacturer's manual.

### Preparation and identification of exosomes

When BMSCs fused to 70%-80%, they were washed twice with PBS and then incubated in serum-free stem cell medium for 48 h (Gibco, USA). The medium was collected and centrifuged at 300 × *g* for 10 min and then centrifuged at 2,000 × *g* for 10 min at 4° C. The supernatant was collected and filtered using a 0.22 μm filter to remove cell debris. The supernatant was then transferred to an Amicon Ultra-15 centrifugal filter (Burlington, USA) and centrifuged at 4,000 × *g* at 4° C until the volume was reduced to 200 μL. Then, the sample was washed twice by ultrafiltration PBS and ultrafiltered again to 200 μL.

For exosome purification, the medium was applied to a 30% sucrose/D_2_O pad in an Ultra-Clear^TM^ tube (Beckman Coulter, USA) and centrifuged at 100,000 × *g* for 60 min at 4° C using an optima L-100 XP Ultracentrifuge (Beckman Coulter, USA). Partially purified exosomes were recovered using an 18 g needle, diluted with PBS, and centrifuged through the filter unit at 4,000 ×*g* at 4° C until the final volume reached 200 μL. The solution was stored at -80° C or used immediately for the experiment. The concentration and size distribution of exosomes were measured using Nanosight Tracking Analysis (NTA). Transmission electron microscopy (TEM) was used to identify the morphology of exosomes. Exosome surface marker proteins, including CD81, CD9 and CD63 were analyzed by western blot.

### Exosomes uptake

To fluorescently label exosomes, Dil solution (Eugene, USA) was added to PBS and incubated with exosomes according to the manufacturer's instructions. Excess Dil dye was removed by ultracentrifugation at 100,000 × *g* for 1 h at 4° C. The pellet was washed three times with PBS and resuspended. The Dil-labeled exosomes (Dil-exosomes) were incubated with RAW264.7 cells for 24 h. Then, cells were washed with PBS and fixed in 4% paraformaldehyde, and the uptake of Dil-exosomes was observed using laser confocal microscopy. Dil-exosomes were also injected into the joint cavity of model rats (described below). After 2 h, the rats were anesthetized and the knee joint was removed for the preparation of sections. Sections were stained with 4’,6-diamidino-2-phenylindole (DAPI) and observed under a fluorescence microscope.

### Incubation of exosomes and RAW264.7 cells

The murine macrophage RAW264.7 cell line was purchased from the American Type Culture Collection (ATCC) and cultured in HG-DMEM medium containing 10% FBS and 1% P/S. The cells cultured in that medium were defined as M0 macrophages. M0 macrophages were incubated with 50 ng / ml LPS (Beyotime, China) or 50 ng/mL LPS + 1 μg/mL BMSC-derived exosomes for 24 h. Cells were harvested after centrifugation and macrophage polarization was identified using flow cytometry and immunofluorescence. The supernatant was collected and tested for cytokine expression levels.

### Co-culture of RAW264.7 cells and chondrocytes

RAW264.7 cells were co-cultured with chondrocytes utilizing Transwell cell culture inserts (Corning, USA) with a 0.4 μm pore sized membrane. Briefly, RAW264.7 cells were seeded on the upper chamber of the co-culture system with 50 ng/mL LPS or 50 ng / ml LPS + 1 μg / ml BMSC-derived exosomes for 24 h. Then, the RAW264.7 cells were washed three times with PBS and chondrocytes were seeded into the lower chamber of the co-culture system. RAW264.7 cells were then co-cultured with chondrocytes for 72 h, after which chondrocytes were collected and the phenotype was identified by western blotting and immunofluorescence.

### Western blot analysis

Total protein was extracted from cells and tissues, and the protein concentration was determined using a BCA assay kit (Beyotime, China). SDS loading buffer (5X) was added to the protein sample, boiled for 10 min to denature the proteins, and stored at -20° C or used immediately for testing. The protein sample was loaded onto an SDS-PAGE gel and transferred to a 0.22 um PVDF membrane by electrophoresis. After membrane transfer, the PVDF membrane was incubated with blocking solution (Beyotime, China) for 2 h at room temperature and then incubated with the following primary antibodies overnight at 4° C (Abcam, USA): anti-Collagen X antibody, anti-Collagen II antibody, anti-Runx2 antibody, anti-SOX9 antibody, and anti-GAPDH antibody. After washing three times with TBST for 10 min each time, the membrane was incubated with horseradish peroxidase-conjugated secondary antibody (Abcam, USA) for 1 h at room temperature. Blots were detected using an enhanced chemiluminescence kit (Beyotime, China).

### Cell fluorescence analysis

Cells were washed with PBS and fixed in 4% paraformaldehyde for 15 min, permeabilized with 0.2% Triton X-100 for 20 min, and blocked with PBS containing 3% bovine serum albumin (BSA, Yeasen, China) for 1 h. Then, cells were incubated overnight with the following primary antibodies: anti-F4/80 antibody (eBioscience, USA), anti-iNOS antibody (Abcam, USA), anti-ARG1 antibody (Proteintech, China), anti-Collagen X antibody (Abcam), USA), and anti-Collagen II antibody (Abcam, USA). Alexa 488 or Alexa 594 dye-labeled secondary antibodies (Jackson, USA) were added and incubated for 1 h at room temperature. Nuclear staining was performed for 5 min by the addition of 4',6-diamidino-2-phenylindole (Beyotime, China) solution. Immunoreactivity was visualized using a fluorescence microscope (Carl Zeiss, Germany).

### Flow cytometry analysis

Cells were washed with PBS and incubated with 3% BSA for 1 h to block non-specific antigen binding. Cells were resuspended after centrifugation and incubated with the following fluorescein-conjugated antibodies for 30 minutes at 37° C in the dark: FITC anti-CD44 antibody (BD Biosciences, USA), PE anti-CD90 antibody (BD Biosciences, USA), PE anti-CD45 antibody (BD Biosciences, USA), PE anti-CD11b antibody (BD Biosciences, USA), APC anti-CD206 antibody (eBioscience, USA), and FITC anti- CD11c antibody (BD Biosciences, USA). Cells were then collected and washed with PBS after centrifugation. Fluorescence was evaluated by BD Cyan flow cytometry (BD Bioscience, USA) and further analysis was performed using FlowJo software (Tree Star Inc., USA).

### Toluidine blue staining

To observe secreted GAG in the cartilage matrix, chondrocytes cultured in the co-culture system were washed with PBS and fixed in 4% paraformaldehyde for 15 min, then stained with toluidine blue (Solarbio, China), according to the manufacturer's instructions. The results with an optical microscope (Leica, Germany).

### OA model and experimental group

Healthy adult male Sprague–Dawley rats (weighing 200−250 g) were purchased from the Animal Center of Nanjing Medical University (Nanjing, Jiangsu, China). In the animal facility, all animals were maintained under specific pathogen-free (SPF) conditions. The experimental procedure was approved by the Animal Care and Use Committee of the Institutional Laboratory of Nanjing Medical University, and maximized attention to limiting the number of animals used in this study. The OA model was induced using the modified Hulth technique. Chloral hydrate (10%; 0.1 mL/100 g) was intraperitoneally injected into the anesthetized rats, the right knee joint was taken longitudinally 2 cm, the anterior cruciate ligament was cut, the medial meniscus was removed, sutured layer by layer and intramuscular injection of penicillin 2 × 10 U/kg per day for a total of 5 days. All rats undergoing surgery or sham surgery were randomly divided into three groups (n=12 in each group): (1) Sham group; (2) PBS group; (3) BMSC-derived exosomes group. At 4 weeks post-operation, rats were injected intra-articularly with 10 ul of PBS or BMSC-derived exosomes (10^10^ particles/ml) using a microsyringe for 3 days for 4 weeks.

### Micro CT analysis

Knee joint samples were fixed in 4% paraformaldehyde and micro computed tomography (Micro CT) of the fixed knee joint specimens was performed using a photomicrography imaging system (ZKKS MCT Sharp III scanner, China). The small field was selected for scanning and correcting the CT value with a 70kV scan voltage, 30 W power, 429 μA current, and 5 μm scan thickness. 3D-Matic Research 11.0 software was used for 3D knee reconstruction and image capture. The region of interest (ROI) was selected from the periarticular epiphyses using Mimics Research 19.0 software. The ROI size was blindly calculated on all four condyles of the knee (the medial and lateral sides of the tibia and femur) and the mean was used for statistical analysis.

### Histological analysis

The knee joint was fixed in 4% paraformaldehyde, decalcified in 20% formic acid, and embedded in paraffin. By collecting 5 μm sections at intervals of 50 μm, continuous sagittal sections, including the entire joint were obtained. Sections were stained with Safranin O-fast green and HE for histological analysis. The middle section was used for immunohistochemistry and immunofluorescence analysis. Synovial activation was assessed by scoring the cell thickness of the synovial lining (0-3) using HE stained slides [37, 38]. The medial and lateral compartments of the joint were scored separately and given the sum of the two scores (maximum site score 6). Cartilage degeneration was graded in Safranin-O/Fast Green-stained sections using the Mankin criteria, modified by the International Association of Osteoarthritis Research (OARSI).

### Immunohistochemistry and immunofluorescence

Samples were prepared as previously described. After dewaxing and rehydration, the sections were soaked in citrate buffer at 60° C overnight to reveal the antigen. For immunohistochemical staining, we added 3% hydrogen peroxide for 10 min to inactivate endogenous peroxidase activity, and the sections were blocked with 1% goat serum for 1 h at 37° C, and incubated with the following primary antibody overnight at 4° C: anti-Collagen X antibody (Abcam, USA) and anti-Collagen II antibody (Abcam, USA). Samples were then stained with horseradish peroxidase-conjugated secondary antibody (Abcam, USA). Then, 3,3-diaminobenzidine was observed for chromogen and counterstained with hematoxylin. For immunofluorescence, the sections were incubated with primary antibody (anti-ARG1 antibody (Proteintech, China) overnight at 4° C, and then stained with Alexa 488 or Alexa 594 dye-labeled secondary antibody. Nuclei were labeled with DAPI and images were obtained using a fluorescence microscope.

### Cytokine determination

The synovial fluid samples of OA rats and the supernatant samples of RAW264.7 cells were collected. Cytokine levels were measured using the following ELISA kits, according to the manufacturer's protocol (All from Abcam, USA): Mouse IL-6 ELISA Kit, Mouse IL-1 beta ELISA Kit, Mouse TNF alpha ELISA Kit, Mouse IL-10 ELISA Kit, Rat IL-6 ELISA Kit, Rat IL-1 beta ELISA Kit, Rat TNF alpha ELISA Kit, and Rat IL-10 ELISA Kit. The results were tested using a multifunctional enzyme marker.

### Statistical analysis

IBM SPSS Statistics v20.0.0 software was used to process and analyze the data and images. Data are expressed as the mean ± SEM. Statistical differences were assessed using the Student's *t*-test, or one-way or two-way ANOVA. A P-value <0.05 was considered significant.

### Ethics approval

All animal experiments were approved by the Animal Care and Use Committee of the Institutional Laboratory of Nanjing Medical University (30^th^ 2019 with ethics code: IACUC-1903035).
